# New imaging tools reveal live cellular collagen secretion, fibril dynamics and network organisation

**DOI:** 10.1038/s41598-025-96280-4

**Published:** 2025-04-21

**Authors:** Olivia Kent, Eleanor R. Casey, Max Brown, Steven Bell, Matthew C. Ehrman, Michael J. Flagler, Arto Määttä, Adam M. Benham, Timothy J. Hawkins

**Affiliations:** 1 Department of Biosciences, Durham University, South Road, Durham, DH1 3LE UK; 2 The Procter & Gamble Company, Newcastle Innovation Centre, Newcastle-Upon-Tyne, NE12 9TS UK; 3 Procter & Gamble International Operations SA SG Branch, 70 Biopolis Street, Singapore, 138547 Singapore; 4 The Procter & Gamble Company, Mason, OH 45040 USA

**Keywords:** Cellular imaging, Microscopy, Fluorescence imaging, Imaging, Time-lapse imaging, Cell biology, Extracellular matrix

## Abstract

Although light microscopy has been used to examine the early trafficking of collagen within the cell, much of our understanding of the detailed organisation of cell deposited collagen is from static electron microscopy studies. To understand the dynamics of live cell collagen deposition and fibril organisation, we generated a bright photostable mNGCol1α2 fusion protein and employed a range of microscopy techniques to follow its intracellular transport and elucidate extracellular fibril formation. Our findings reveal the dynamics of fibril growth and the dynamic nature of collagen network interactions at the cellular level. Notably we observed molecular events that build network organisation, including fibril bundling, bifurcation, directionality along existing fibrils, and looping/intertwining behaviours. Strikingly, mNGCol1α2 fluorescence intensity maxima can mark a fibril before another growing collagen fibril intersects at this location. Real-time, high-resolution imaging of collagen has enabled fibrillogenesis and organisational dynamics to be visualised together in an actively secreting cellular system. We also show that the N-terminal protease site is not an absolute requirement for collagen fibril incorporation. This approach paves the way for assessing the dynamic organisation and assembly of collagen into the extracellular matrix in skin models and other tissues during health, ageing and disease.

**Supplementary Information:**

The online version contains supplementary material available at 10.1038/s41598-025-96280-4.

## Introduction

Collagens are essential extracellular matrix (ECM) proteins which account for approximately 30% of the total protein mass of mammals^1^. These collagens are diverse, comprising 28 fibrillar and non-fibrillar proteins, expressed in connective tissues including the skin, bone, and cartilage^1^. Collagen proteostasis is sensitive to both oxidative and reductive stress^2^. Disruption to healthy collagen structure or expression is a significant factor in fibrosis, osteoporosis and several genetic diseases^1,3^ but is also a natural consequence of skin aging. For example, the *COL1A1* type I collagen is significantly down-regulated with age across photo-exposed and non-photo-exposed skin sites^4^. Type I collagen, which accounts for 90% of human collagen, is comprised of two α1 chains (expressed by the *COL1A1* gene) and one α2 chain (expressed by the *COL1A2* gene) that associate as a triple helix heterotrimer to form a procollagen polypeptide of approximately 300 nm in length^5^. This heterotrimer assembles in the ER under the guidance of multi-functional chaperones, such as PDI (P4HB), P4HA, and the collagen-specific chaperone Hsp47^6–8^. Procollagen undergoes extensive processing before and after secretion from the cell, including N- and C- terminal proteolytic processing and fibrillogenesis^9^. Although collagen egress from the ER and ER-Golgi transport^10^ has been extensively studied, less is known about the post-Golgi transport of collagens to the plasma membrane (PM) and the rate determining steps of collagen secretion in different cell types*.* Following secretion, collagen is deposited into the ECM where, facilitated by fibronectin, it self-assembles into a fibrous network to provide connective tissues with mechanical strength^11,12^.

Although electron microscopy can inform us about the complex organisation of preassembled collagen networks surrounding cells and tissues, dynamic information about how collagen fibrils elongate and are organised by living cells to generate these networks is limited. By pairing a novel bright and stable collagen fusion protein with advanced high temporal and spatial microscopy techniques, we have charted the trafficking and assembly of collagen; capturing individual secretion events and observing the live-cell dynamics of collagen deposition, fibril growth and organisation from live cells. Overall, the study reveals how dynamic phenomena establish network organisation at the cellular level.

## Results

### Construction of an mNG-type I collagen fusion protein marker, mNG-Col1α2.

**Fig. 1:**
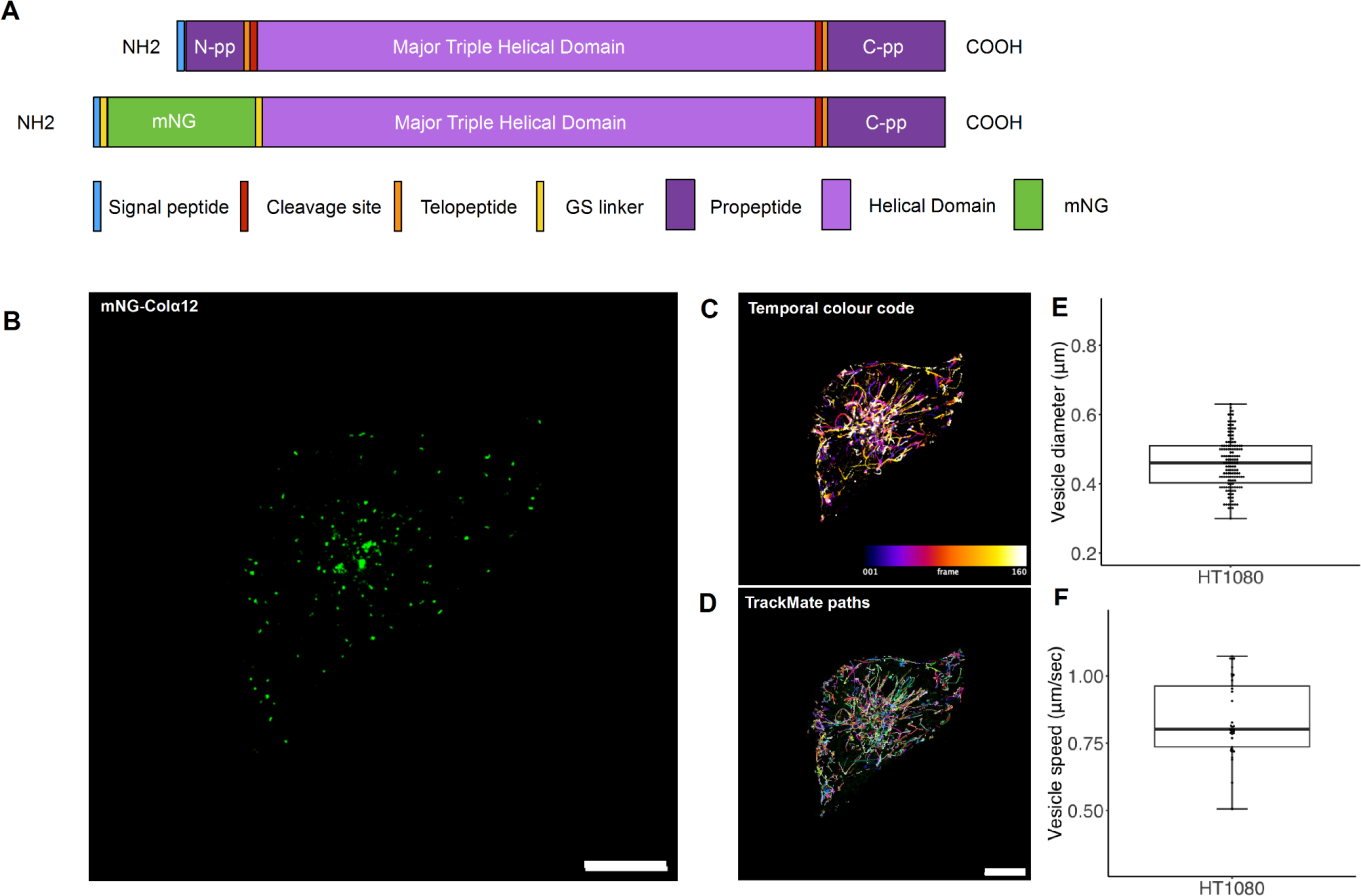
Live cell imaging of mNGCol1α2 expressed in HT1080 cells. (**A**) Diagram of mNG-Col1α2 fusion protein. (**B**) HT1080 cells expressing mNG-Col1α2. mNG-Col1α2 highlights small rapidly moving particles which predominantly move towards the periphery of the cell. (**C**) Time coding projection illustrates passage of particles towards the periphery. (**D**) Particles were isolated and tracked using TrackMate and Fiji to calculate size, number and velocity. Scale = 10 μm, 206.44 ms per frame or 4.844 fps. (**E**) Combined box and whisker and beeswarm plots of HT1080 collagen vesicle diameter showing all data points. Mean diameter = 0.47, standard deviation = 0.07 n = 150 (3 replicates per cell, 10 cells per replicate, 5 vesicles per cell. (**F**) Combined box and whisker and beeswarm plots of HT1080 collagen vesicle speed showing all data points. Mean speed = 0.84 μms^−1^ Standard deviation = 0.14 μms^−1^, n = 46.

To follow the trafficking, exocytosis and fate of type I collagen at high spatial and temporal resolution we created a fusion protein combining the bright and stable fluorescent protein mNeonGreen (mNG) with the Col1α2 chain. mNG, a green/yellow fluorescent protein isolated from the marine invertebrate *Branchiostoma lanceolatum,* is ~ 4 fold brighter and more photostable than GFP, allowing it to be detected by lower laser intensities, over longer periods and at a higher frame rate^13–15^. A similar fusion protein strategy, employing an N-terminal GFP instead of mNG, has been used to observe collagen in vitro^16^, and at the tissue level in a zebrafish model^17^. Tagging Col1α2 is preferable to Col1α1 because two tagged Col1α1 monomers within the heterotrimeric complex could increase the possibility of steric effects and produce variation in labelling intensity.The fusion protein in this study retains the N terminal signal peptide, replacing the N-propeptide (pp) with mNG, which is flanked by GS linkers to confer flexibility (Fig. 1A). Importantly, removal of the N terminal ADAM proteinase site prevents proteolysis of the mNG tag on collagen following secretion of the fusion protein into the ECM enabling the observation of fibrillogenesis.

The mNG-Col1α2 fusion was tested first in HT1080 fibrosarcoma cells, which correctly assemble and quality-control collagens but lack endogenous expression of Col1^18^, enabling mNG-Col1α2 to be evaluated in the absence of competitor Col1 chains.

### mNG-Col1α2 traffics from the ER, interacts with expected folding partners and becomes hydroxylated.

To confirm that mNGCol1α2 was correctly processed and post-translationally modified, mNG-Col1α2 was immunoprecipitated (IP) from transfected HT1080 cells and subjected to data-dependent acquisition mass spectrometry (DDA MS). mNG-Col1α2 interacted with key processing proteins including P4HA and P4HB (PDI) (Supplementary Figure 1A)^19^. To confirm the integrity of the fusion protein, the peptide sequences of the trypsin-digested immunoprecipitates were assessed by protein pilot. Peptide coverage spanned the entire fusion protein, and mNG-Col1α2 was hydroxylated at multiple sites (Supplementary Figure 1B), demonstrating that the fusion protein could receive specific post-translational modifications in HT1080 cells.

To confirm mNG-Col1α2 trafficking, the exit of proteins from the ER was prevented by treatment with Brefeldin A (BFA). Prior to treatment, mNG-Col1α2 was distributed between the ER, a post-ER compartment likely to be the ERGIC, and small vesicles (Supplementary Figure 1C). Following treatment, high levels of colocalisation of mNG-Col1α2 with the ER resident protein PDI (P4HB) were observed, indicating retention (Supplementary Figure 1D). A statistically significant increase in colocalisation was quantified using the Coloc2 plugin for Fiji. BFA treated cells show a Pearson’s R value of 0.96 compared to 0.26 for control cells, indicating significant colocalisation between mNG-Col1α2 and PDI (Supplementary Figure 1D). Following BFA treatment, peripheral vesicles were lost, consistent with the expectation that newly synthesised collagen is trafficked from the ER to these carrier vesicles.

### mNG-Col1α2 is packaged into highly dynamic collagen carriers

In transfected HT1080 cells the mNG-Col1α2 fusion protein was localised to numerous punctae within the cytoplasm consistent with vesicles (Fig. 1B). The spherical carriers have a mean diameter of 0.47 μm (SD) 0.07 μm n = 150 (Fig. 1E, F). The peripheral location of these structures suggests that they represent Golgi to PM carriers (GPCs). The collagen carriers were highly dynamic with movement principally towards the plasma membrane, although objects moving away from the PM were also observed (Supplementary movie 1). Temporal colour coding (Fig. 1C) was used to demonstrate the trajectory of vesicles with the white latter frames showing vesicles accumulating at the periphery of the cell, often within cellular projections. Motile vesicles were tracked in time-lapse movies using ImageJ and TrackMate. A representative dataset with particle tracking is shown in (Fig. 1D and Supplementary movie 2). Within just 60 s the observed particles transversed the cell, with a mean velocity of 0.83 μms^−1^ (SD 0.14 μms^−1^ n = 46). The enhanced mNG signal intensity allowed short exposures and high frame rates (10–206 ms per frame) enabling accurate capturing of rapid movement without blurring or gaps in the vesicles path (Fig. 1C, Supplementary movie 1).

### Transport of mNG-Col1α2 collagen carriers to PM requires microtubules.

**Fig. 2:**
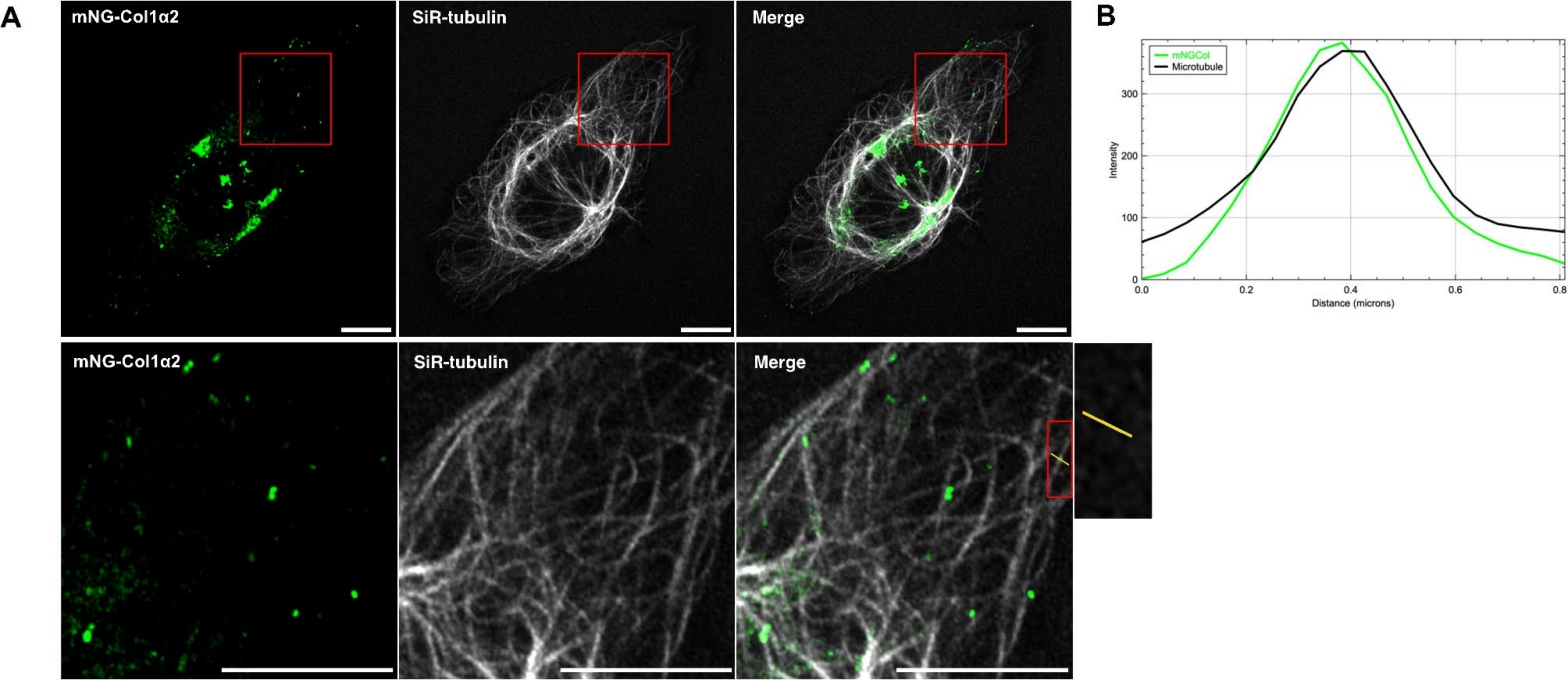
mNG-Col1α2 containing vesicles associate with and move along the microtubule network. (**A**) Live cell imaging of mNG-Col1α2 with SiR-tubulin in HT1080 cells. MNG-Col1α2 particles align along microtubules and move along these paths. Scale = 10 nm in both full cell and zoomed images. (**B**) Quantitative line profile of colocalisation along the yellow line in A.

To further investigate the dynamics of collagen carriers, we studied the microtubule dependency of vesicle movements. Early studies reported that disruption of microtubules by colchicine impairs collagen secretion from fibroblasts^20^. Co-labelling of microtubules in HT1080 cells expressing mNG-Col1α2 revealed that the majority of collagen carriers associate with microtubules (Fig. 2A, B, Supplementary movie 3). High speed super-resolution Airyscan confocal microscopy allowed these small but well-defined carriers/vesicles to be seen clearly, tracking along the microtubule (Supplementary movie 3). Following the disruption of the microtubule network with the polymerisation inhibitor colchicine, these vesicles no longer exhibited directional movement along microtubules. (Supplementary movie 4 & 5).

### High speed TIRF microscopy reveals the fate of collagen containing vesicles

**Fig. 3:**
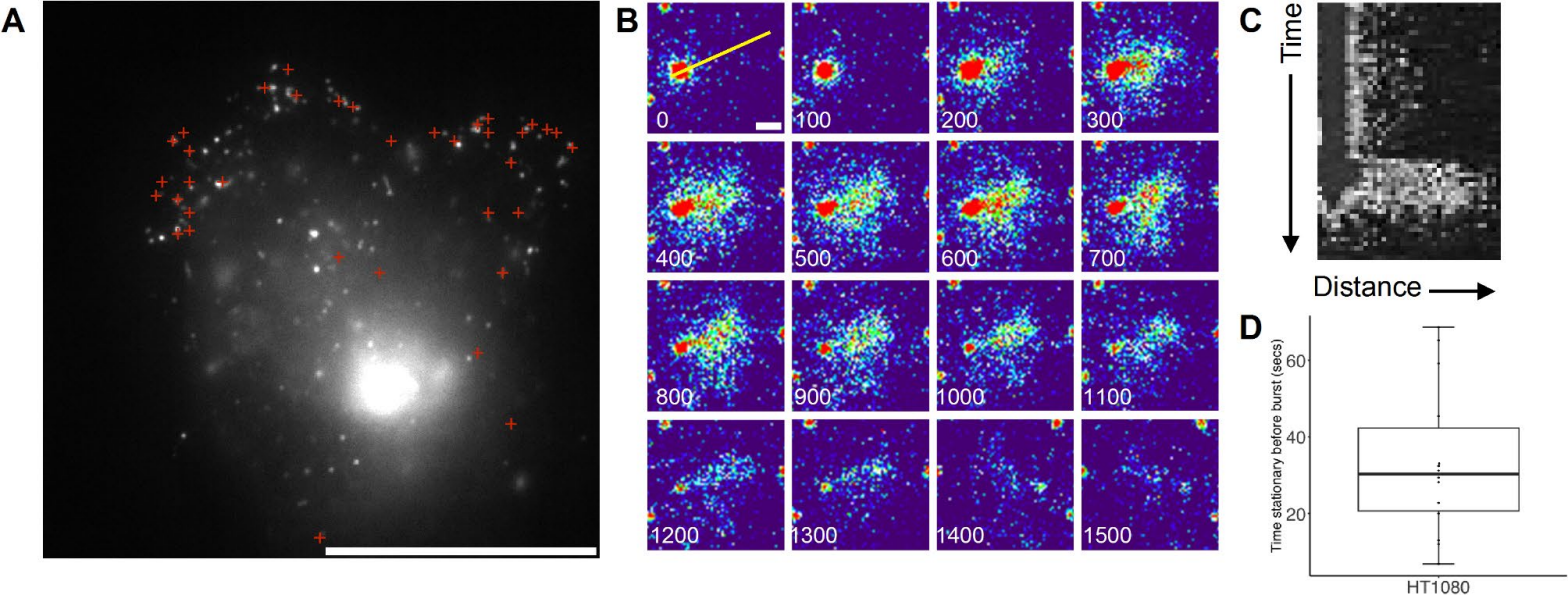
mNGCol-1α2 secretion events. (**A**) Full field of view TIRF image of an mNG-Col1α2 expressing HT1080 cell. Red + mark locations of collagen ‘burst’ events. These events usually clustered at the periphery of the cell on the lower surface. Scale = 20 μm. (**B**) TIRF movie frames of a burst event. 100 ms between frames. Scale = 1 μm. (**C**) Kymograph of burst event derived from yellow line in B. (**D**) Box and whisker plot of stationary phase of vesicles at the PM before they fuse with PM to give a burst event.

Having observed trafficking of mNG-Col1α2 through the secretory pathway towards the PM, we used mNG-Col1α2 to study the less understood process of collagen exocytosis. Total Internal Reflection Fluorescence Microscopy (TIRF) produces a thin < 200 nm excitation field at the glass water interface allowing real time visualisation of objects at the PM. The technique has been used to provide insights into the movement of organelles and single molecules at the PM including the moment of exocytosis of synaptic vesicles containing neurotransmitters into the synaptic cleft^21^. We used ring TIRF microscopy to follow the vesicles at high temporal resolution (10–100 ms). Multiple bursts occurred at the PM within the 200 nm TIRF field which represent the moment of exocytosis. Bursts mostly occurred at the Baso-lateral surface of the cell predominantly towards the lateral periphery (Fig. 3A). Motile carriers approaching the PM stopped, becoming static for a short period, before ‘bursting’ and releasing the mNG-Col1α2 cargo (Fig. 3B). The high frame rate resolves the release and subsequent dispersal of the fluorescent signal (Fig. 3B, C and Supplementary movie 6). Observed pausing events of a mean duration of 33.4 s (SD 19.6) n = 14) may represent the capture of the carrier by the actin/myosin cytoskeleton and docking at the PM^22^ (Fig. 3D). Intriguingly in the example shown (Supplementary movie 6) with 10 ms temporal resolution, the carrier appears to transition past an exocytosis location and return, before becoming stationary and releasing its contents.

### Super-resolution imaging of extracellular deposited mNG-collagen fibrils from live cells.

**Fig. 4:**
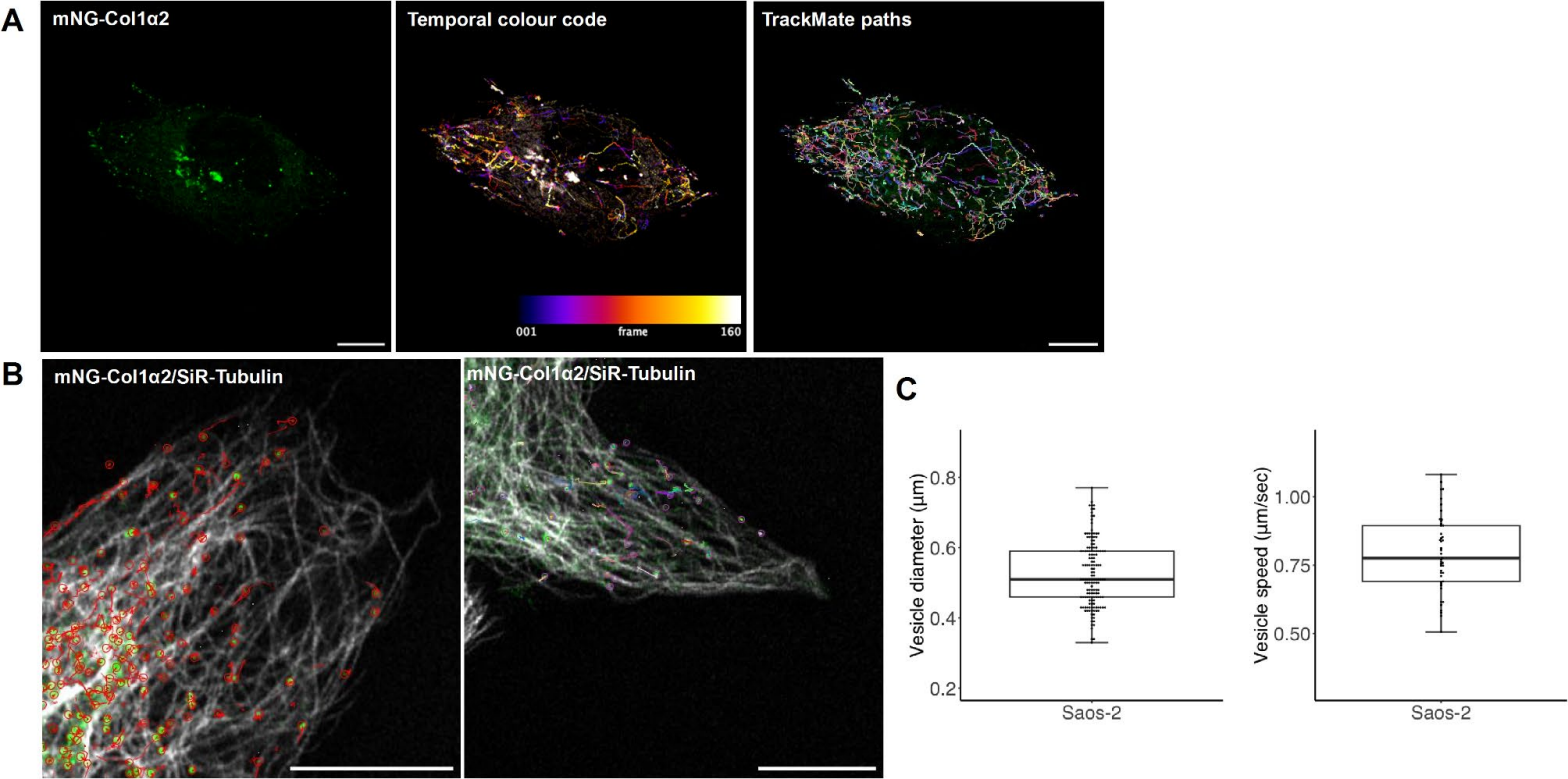
mNG-Col1α2 labels fast-moving carriers in Saos-2 cells with movement along microtubules. (**A**) mNG-Col1α2 labels collagen carriers or vesicles which rapidly move towards the periphery. These paths are shown by temporal colour coding. TrackMate tracks are used to quantify vesicle size and velocity. Scale = 10 µm. (**B**) Saos-2 cells expressing mNG-Col1α2 co-imaged with microtubules labelled by SiR-tubulin. Overlay shows mNG-Col1α2 association with microtubule. Superimposed on these images are the paths of TrackMate detected paths of mNG-Col1α2 carriers demonstrating their movement along microtubules. (**C**) Combined box and whisker and beeswarm plots of Saos-2 collagen vesicle diameter showing all data points. Mean diameter = 0.53 μm, standard deviation = 0.09 μm n = 150 (3 replicates per cell, 10 cells per replicate, 5 vesicles per cell). Combined box and whisker and beeswarm plots of Saos-2 collagen vesicle speed showing all data points. Mean speed = 0.79 μms^−1^, standard deviation = 0.14 μms^−1^ n = 45.

**Fig. 5:**
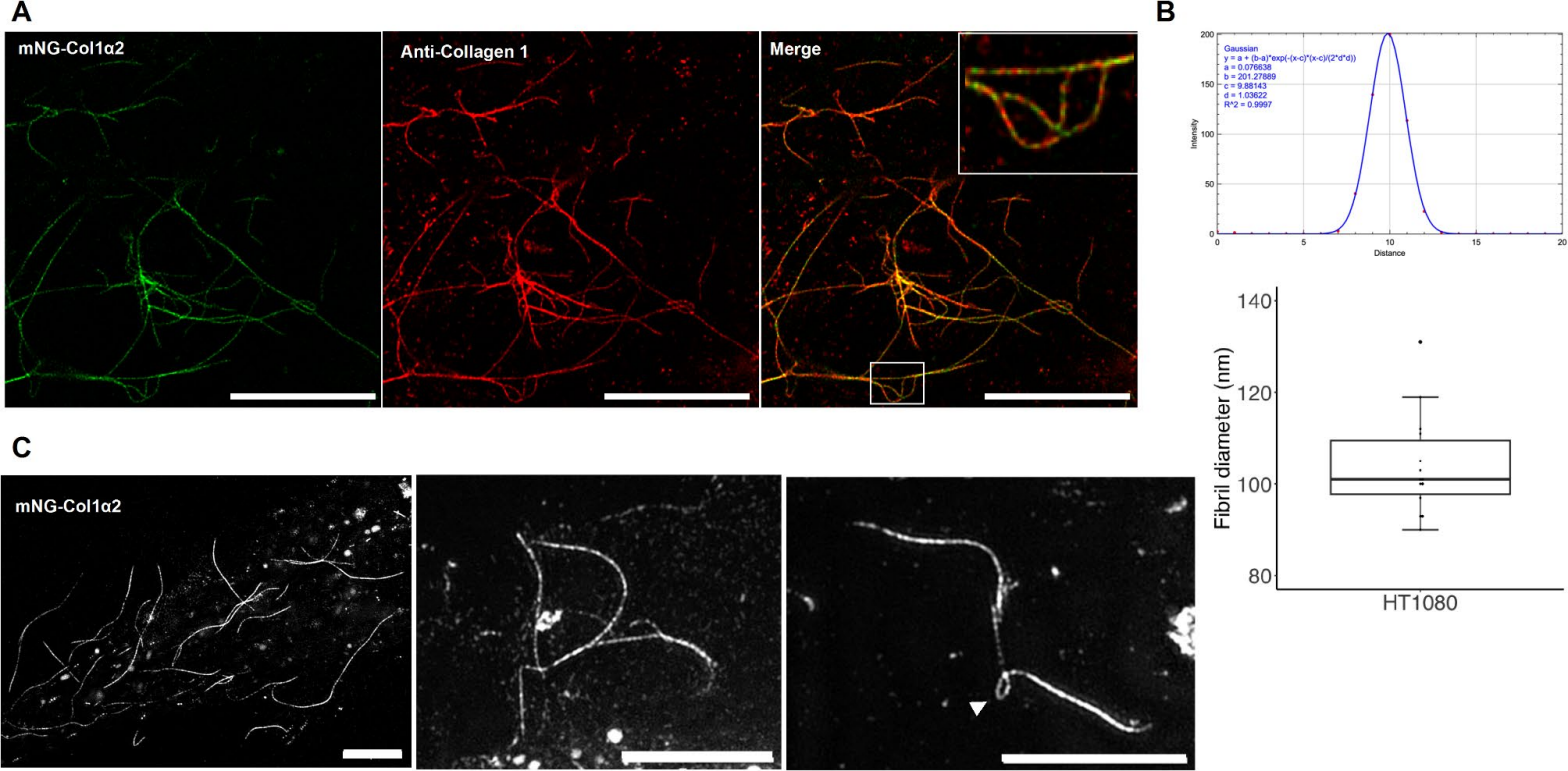
Superresolution microscopy of collagen fibrils containing mNG-Col1α2 deposited by Saos-2 cells. (**A**) Airyscan jDCV Super-resolution image of deposited mNG-Col1α2 collagen fibrils (green). Fibrils are co-labelled with anti-Collagen 1 antibody (red). In the merge panel areas of colocalisation along the fibril appear yellow. Zoomed area shows the relationship between accessible native Col1 epitopes and the incorporated mNG-Col1α2. Scale = 10 μm. (**B**) FWHM analysis of deposited collagen in A. Box and whisker plot shows range and of collagen diameter values. SD = 12.53 n = 32. (**C**) 3DSIM super-resolution imaging of deposited mNG-Col1α2 fibrils. These three-dimensional stacks reveal loops within fibrils. Scale = 5 μm.

To investigate the process of collagen fibrillogenesis and deposition of fibrils within the ECM we turned to Saos-2 osteosarcoma cells expressing mNG-Col1α2. Unlike HT1080, Saos-2 cells express both type 1 Col1α1 and α2 chains, and highly express the lysyl hydroxylase LH1 to support the extra-cellular cross-linking of collagen^23^. Transfected Saos-2 cells produce labelled collagen carrier vesicles with a diameter of 0.53 μm (SD 0.09 μm n = 150) and a velocity of 0.79 μms^−1^ (SD 0.14 μms^−1^ n = 45) (Fig. 4A, C, Supplementary movie 7), comparable to those seen in HT1080 cells. These vesicles also move along microtubules, demonstrating that type 1 collagen produced by different cell types utilises the same post-ER transport machinery (Fig. 4B and Supplementary movies 8 and 9). TrackMate analysis was used to chart these vesicle movements along microtubule tracks and colchicine treatment stopped these movements, as seen with HT1080 cells (Supplementary movies 10 & 11). BFA treatment of Saos-2 cells also caused the loss of collagen carriers and accumulation of the collagen signal within the ER (Supplementary Figure 2). To examine the biosynthesis of collagen fibrils outside the cell, Saos-2 cells were cultured in the presence of ascorbic acid for 48 h on fibronectin-coated coverglass-bottomed dishes and imaged with super-resolution microscopy (Airyscan and 3D-SIM). To date, dynamic super-resolution imaging of deposited collagen has not been documented in the published literature. This experiment revealed the first dynamic observation of deposited collagen from live cells at 100 nm resolution and clarity. Fibrils containing mNG-Col1α2 were deposited beneath the cell, linked to the cell, or as a network close to a cell. When imaged with Airyscan with jDCV processing, the collagen fibrils had a mean Full Width Half Maximum (FWHM) of 106 nm (range 91–131 nm SD = 12.53 n = 32) (Fig. 5A, B) consistent with observations of collagen fibril diameters of 35–110 nm in electron microscopy studies^24,25^. This diameter can vary, depending on tissue type, up to ~ 300 nm. These mNG-Col1α2 containing fibrils were readily detected by an antibody that recognises native triple helical type I collagen (Fig. 5A). Both straight and curved fibrils were within the size range expected. Importantly, these results show the integration of mNG-Col1α2 into the lattice of unlabelled native collagen to generate a singular fibril or bundle (Fig. 5A merged insert). Of note, 3D-SIM super-resolution microscopy resolved small loops within individual fibrils in 3D (Fig. 5C). 3D-SIM bleaching experiments showed that the mNG signal did not diminish equally throughout the length of the fibril (Supplementary Movie 12). The distribution of the mNG-Col1α2 bleaching may reflect the number and fine spatial incorporation of the fusion protein with the collagen fibril.

To establish the utility of the imaging platform to compare collagen trafficking and fibril growth across collagen producing cell types, we also confirmed that comparable vesicular carriers and fibril deposition occurred in BJ fibroblast cells expressing mNG-Col1α2 (Supplementary Figure 3).

## Extracellular collagen fibril dynamics

**Fig. 6:**
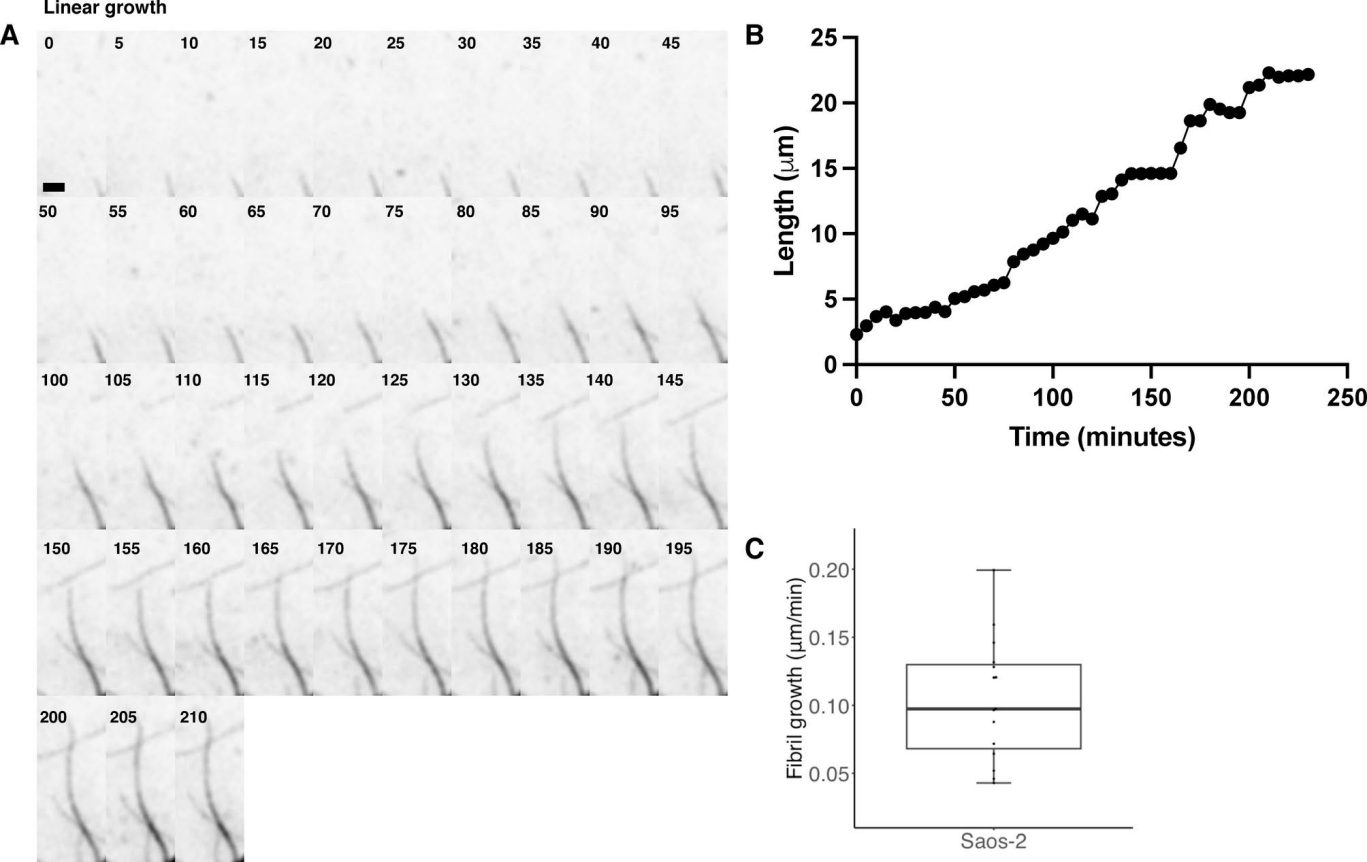
mNG-Col1α2 fibrils exhibit sustained directional linear growth. Frames from a Saos2 mNG-Col1α2 fibril deposition movie (subsection) showing linear growth of a fibril being tracked. (**B**) Graph shows the length of an isolated collagen fibril over time showing the rate of elongation with some pauses and or moments of stalling. Images were acquired at 5 min intervals Frame rate is 12 frames per hour. Time stamps are included on each frame. Scale = 2 μm.

**Fig. 7:**
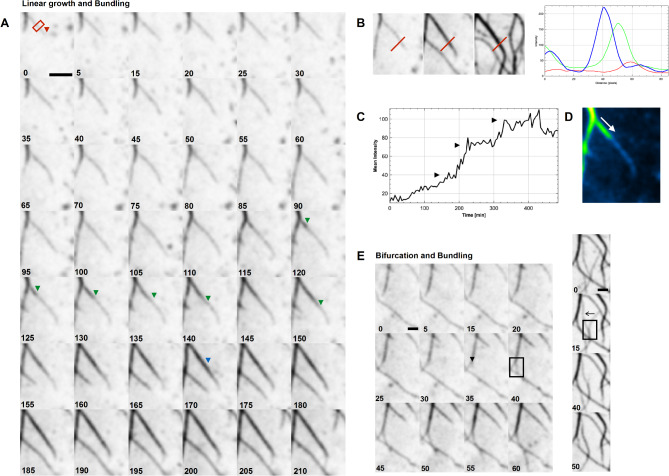
Collagen fibrils can grow along or co-aligned with existing fibrils. (**A**) Frames from an mNG-Col1α2 fibril deposition movie showing growth of collagen fibrils along existing fibrils with potential bundling and increase in signal intensity. Blue, green and red arrows indicate the end of a growing fibril as it passes along an existing fibril observed as a progressive increase in intensity. Scale = 5 μm. (**B**) Three frames taken from movie in A demonstrating three events of “on fibril” or coaligned fibril growth and bundling. Line plots of red line show the intensity and width of the fibril increasing over time (red selected early frame, green selected mid frame and blue selected late frame). (**C**) Time intensity plot of the red box zone in A shows the three events when a fibril travels along the underlying existing fibril. Events marked with a black arrow. (**D**) The direction increase in fibril intensity as a second fibril growth along another is illustrated with the use of colour coding (intensity based Look Up Table). (**E**) Frames from an mNG-Col1α2 fibril deposition movie showing a bifurcation event. Site of bifurcation highlighted by black arrow. The bifurcation event occurs within the black box. Images were acquired at 5 min intervals 12 frames per hour. Time stamps are included on each frame. Scale = 2 μm. (**F**) Selected frames from an mNG-Col1α2 fibril deposition movie showing a zippering event. The zippering occurs within the black box. Black arrow shows the direction of movement/zippering where two separate fibrils start to bundle/zipper from the bottom of the frame snapping together in the direction of the arrow.

**Fig. 8:**
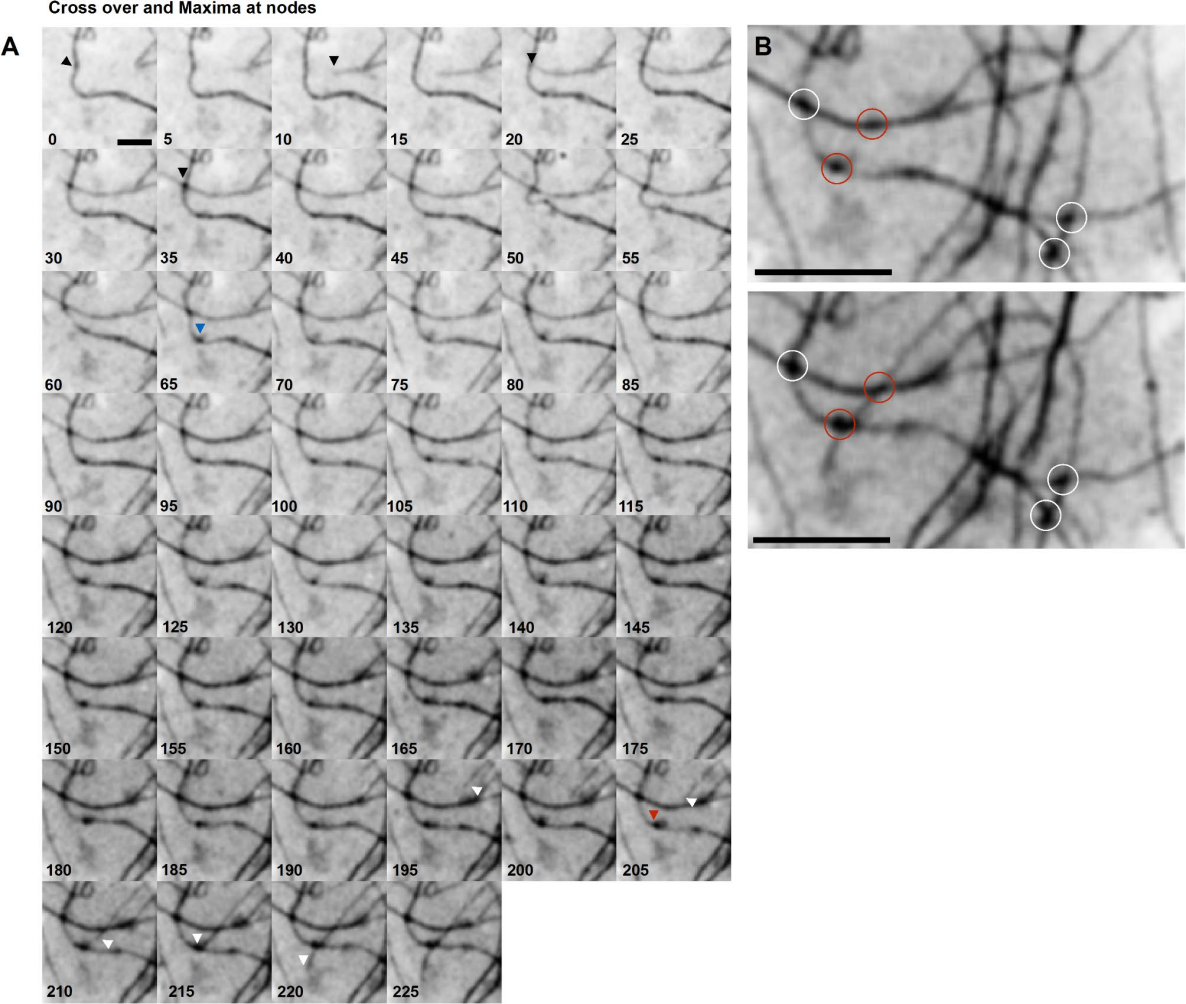
Locations at which collagen fibrils cross over have distinct mNG-Col1α2 fluorescence intensity maxima which can exist before a crossover event. (**A**) Frames from an mNG-Col1α2 fibril deposition movie. Points of fibril-fibril crossover locally increase in intensity following crossover to give dark foci representing a local accumulation mNG-Col1α2 (blue arrows). Black arrows track the end of a fibril which crosses at this point. Alternatively, fibrils can cross over at points where accumulations pre-exist (red arrow). Intersecting fibril tip (white arrow) 5 min per frame. Time stamps are included on each frame. Scale = 2 μm. (**B**) Zoomed in view of crossover event. Dark foci of mNG-Col1α2 accumulation can pre-exist along a fibril. A new growing fibril entering the frame transitions specifically through these points (red circles) suggesting a predetermination of the location of these crossover nodes or fibril growth along a faint unresolved fibril which is already crossing over. Images were acquired at 5 min intervals. 12 frames per hour. Time stamps are included on each frame. Scale = 10 μm.

**Fig. 9:**
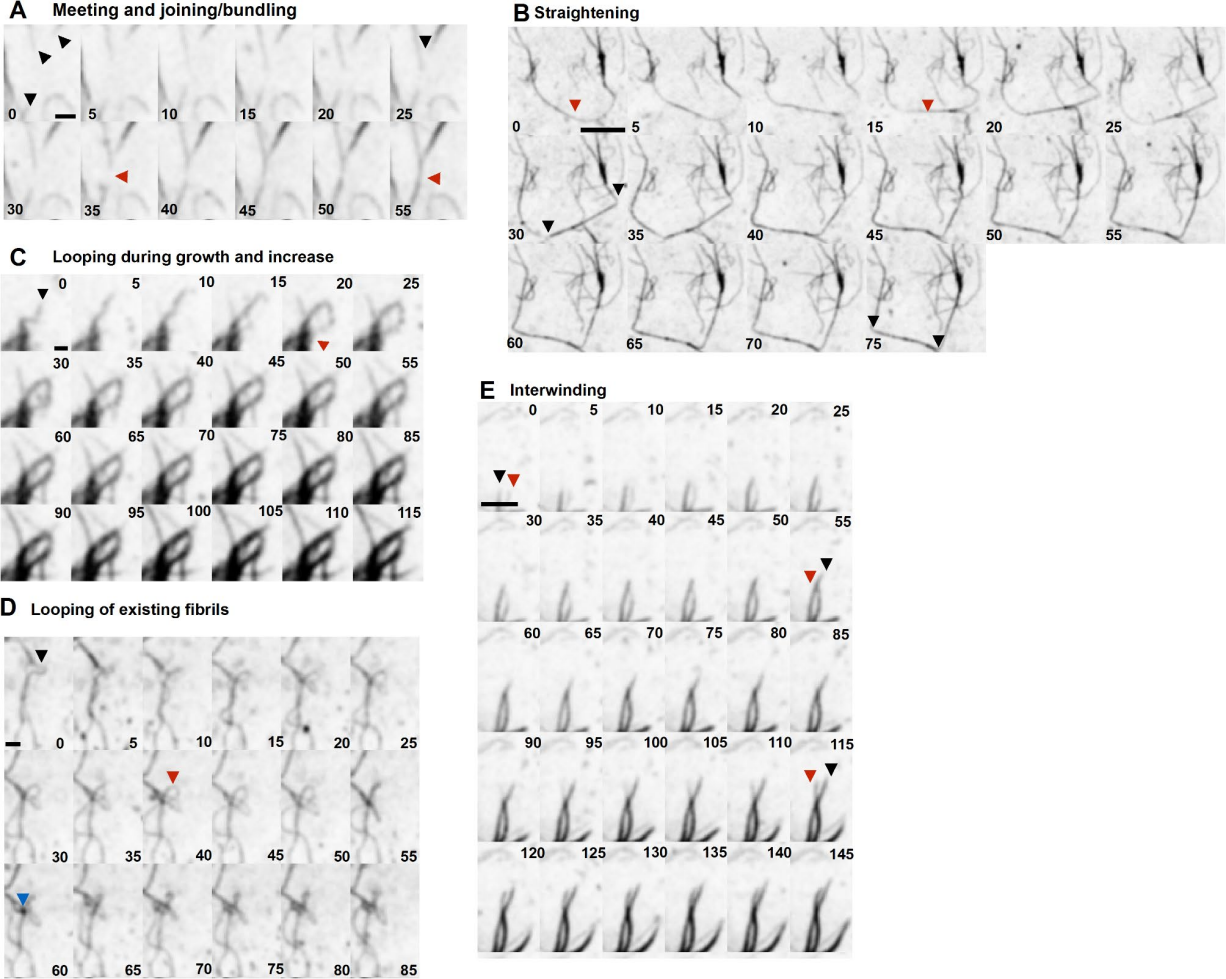
A wide range of further fibril interactions and organisational phenomena are observed. (**A**) Frames from a mNG-Col1α2 fibril deposition movie. Three fibrils highlighted by black arrows grow towards each other before joining at the red arrow. Scale = 2 μm. (**B**) A fibril marked by a red arrow straightens as if under tension. Two corner attachment points are marked by black arrows between which straightening occurs. Scale = 10 μm. (**C**) Growing fibrils produce a loop structure that is further strengthened by addition of more mNG-Col1α2 to give a thick loop. Tip of fibril that loops back indicated by black arrow. Red arrow shows where it rejoins the existing bundle. Scale = 2 μm. (**D**) Existing fibrils can be pulled into loops potentially by flow or mechanical force from the cell above. Black arrow marks the fibril forming a kink. Red arrow markes the established loop. Blue arrow marks a dark punctae of increased collagen signal at the cross over point. Both of these loops could represent further sites of attachment for another motile cell. Scale = 2 μm. (**E**) Frames from a mNG-Col1α2 fibril deposition movie. Two separate mNG-Col1α2 labeled fibrils intertwine around each other followed by an increase in fibril intensity and width. The tip of each growing fibril is marked by a black or red arrow. For all movies the images were acquired at 5 min intervals. 12 frames per hour. Time stamps are included on each frame. Scale = 5 μm.

**Fig. 10:**
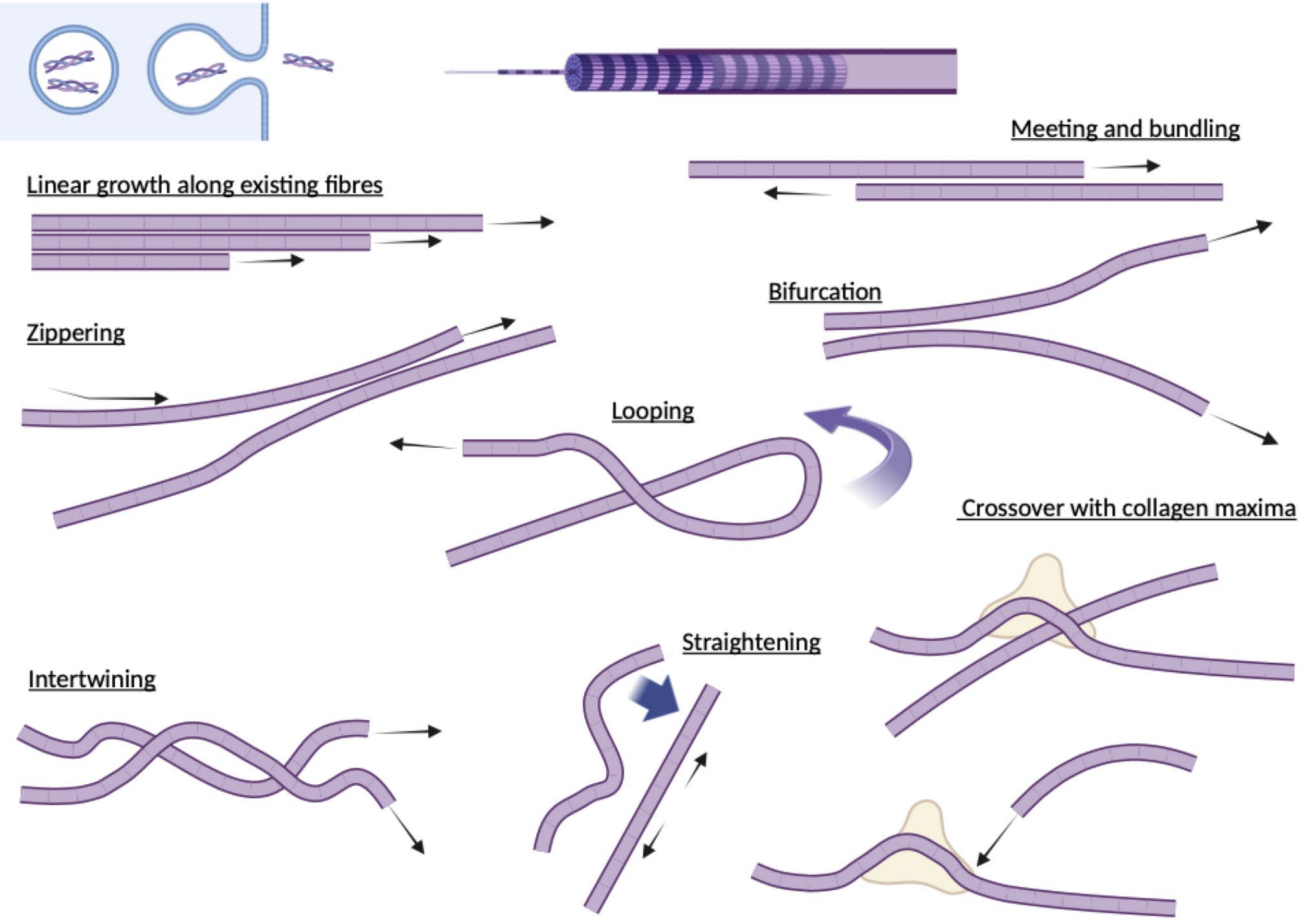
Summary diagram of the observed multiple ways that collagen fibrils can grow and become organised. Created in BioRender.

The deposited collagen network was dynamic even over short timescales of minutes where it was seen to flex, potentially due to new fibril incorporation and attachment, or the motility of the overlying cell. Longer term live cell imaging of mNG-Col1α2 over 18–20 h enabled the observation of extracellular collagen fibril growth and dynamics in unprecedented detail and revealed the existence of a range of fibril dynamic phenomena which have to date not been reported**.** The directional growth of an individual collagen fibril was tracked and found to have an average rate of 0.10 μm min^−1^ (SD = 0.045 n = 15), with these fibrils often radiating from a preexisting bundle (Fig. 6A and Supplementary movie 13). Fibrils followed straight and curved trajectories with periods of both slightly slower and faster growth rates, including pauses, but maintaining a positive trajectory (Fig. 6B). In our experiments negative trajectories, which could potentially indicate filament breakdown or turnover by MMPs, were not observed. In addition, we observed at least six distinct dynamic process or organisational phenomena:

1. *Growth of fibrils along pre-existing fibrils with bundling and bundle bifurcation* –additional fibrils grow along existing fibrils (black arrow follows the tip of this second fibril) (Fig. 7A, Supplementary Movie 14). The fibrils increased in intensity and widened as new fibrils extend along the exiting network, likely indicating packing together (Fig. 7B, C). Monitoring of fluorescence intensity within a defined ROI (red box) shows that the intensity of this zone increased in three steps representing each fibril passing through (Fig. 7D). We observed bundle bifurcation, as the linked fibrils separated to follow different trajectories or started to zipper and snap together (Fig. 7E, Supplementary movie 15).
2. *Fibril crossover—*collagen fibrils grew across existing fibrils, where the increase in collagen signal at nodes suggests collagen reinforcement of the site (Fig. 8A arrows, & Supplementary movie 16). Strikingly, when movie frames are examined closely, the maxima can form before the crossover occurs at that location (red circles), potentially predetermining the site of interaction (Fig. 8B). In some cases, a puncta of collagen forms on an existing fibril and grows in intensity; the approaching fibril crosses at this point and further localised intensification follows the crossover event (Supplementary movie 13).
3. *Meeting and joining*—fibrils growing in opposite directions can meet and join. In these circumstances, the two fibrils appear to move closely past each other and join, potentially through bundling, to form a new thinker and singular bundle (Fig. 9A and Supplementary movie 17).
4. *Straightening*—curved and flexible fibrils can become arrays, likely driven by mechanical forces either exerted by the cell above or through cell migration. These fibrils may also represent growth between two anchor points (Fig. 9B and Supplementary movie 18).
5. *Looping*—fibrils often looped back on themselves during growth (Fig. 9C and Supplementary movie 19), or pre-existing fibrils were looped or pushed up by mechanical forces exerted by the cell. This looped fibril architecture was clearly resolved with 3D-SIM (Fig. 9D, Supplementary movie 20). Once growing fibril loops formed, they could be fortified further with new collagen (Fig. 9C and Supplementary movie 15).
6. *Intertwining*—Individual fibrils or bundles of fibrils can wind around each other with crossovers potentially forming the basis for a larger and thicker accumulation of collagen or a network of collagen. (Fig. 9E and Supplementary movie 21). The multiple ways that collagen fibrils can grow and become organised is summarised in Fig. 10.

## Discussion

In this study, we have captured the sought-after moment of secretion of a collagen molecule with high temporal resolution and have developed a system for imaging live cell fibrillogenesis and organisation.

The terminal mechanisms by which cells direct complex packages of collagen to the ECM remain largely unknown. We show that rapidly moving collagen-containing packages pass along microtubules towards the cell periphery, consistent with the kinesin driven collagen trafficking in myofibroblasts^26^. The collagen carriers are captured at the cell membrane, remaining stationary for ~ 30 s until they fuse with the PM and release their cargo. Further co-labelling studies will be required to identify the molecular mechanism involved, but it is likely to require actin capture or a SNARE based exocytosis processes^22^. Alternatively, additional motor proteins, such as myosins, could be involved to facilitate short distance movement, membrane fusion and secretion. Collagen containing packages may be captured by myosin association with actin at the PM as seen in melanosome exocytosis^27^. Notably, we did not observe the colocalisation of secretion sites and the location of fibril nucleation/growth in our system.

We show that, following secretion from Saos-2 cells, the mNG-Col1α2 fusion protein can produce extra-cellular fibrils and successfully incorporate native type 1 collagen into these fibril arrays, with super-resolution microscopy capable of visualising these sites along a fibril. Our live cell imaging of fluorescent collagen in Saos-2 cells revealed the dynamic nature of collagen assembly, with the fibrils exhibiting a range of organisational interactions. Several of these observations have been predicted from electron microscopy of collagen matrices in tissue, yet this is the first record of these higher order networks occurring and developing in a living cell at the subcellular level. Processing and fibril formation occurs outside the cell, with fibronectin also facilitating procollagen cleavage at the cell surface^28^. We observed the formation of fibrils both at the plasma membrane, baso-lateral to the cell, and extending beyond the cell. These observations are consistent with other studies that suggest that fibril assembly is likely a cell-controlled process occurring at the plasma membrane where the cell surface assists in collagen assembly^29,30^. Whether this surface collagen assembly is facilitated by fibronectin interactions, or integrins directly, is debated^29–31^. Work by Silverman and colleagues, using fibronectin antibodies and an externally applied fluorescent collagen binding protein stain (CNA35), demonstrated that thin cell-to-cell and cell-to-glass membrane extensions, when stretched, can recruit fibronectin. Subsequently, fibronectin recruits collagen “aggregates” which develop into linear arrangements, nucleating and promoting fibril assembly in a force dependent manner^32^. It is likely that a variety of collagen attachments and collagen nucleation mechanisms are at play. It will be interesting to translate our approach to a 3D environment where both lateral and axial cell collagen contacts are involved. Silverman and colleagues have also observed growth events of small, discrete 3 μm, collagen ‘worms’ that form on the underside of the cell including one observation of a short collagen branch. The fibrils tracked in our paper are much longer, extending up to 40 μm and the growth rate of 0.1 μm per min is 5 fold faster with the average rate matching the peak growth rate observed by Silvernman et al. Furthermore, the fibril growth in our system is sustained over a much longer period of 5 h compared to 44 min, resulting in the development of a more complex interconnected network that allows the observation of multiple organisational phenomena. Silverman et al. also saw no continuous filament formation without a contraction event. It is possible that these observations differ in part due to the different labelling techniques (genetic vs external binding protein) and the potential fundamental differences in collagen deposition and organisation between PHCF (primary human corneal fibroblast) cells and Saos-2 (an osteoscarcoma).

The mNG-Col1α2 containing fibrils in our experiments were readily detected by antibodies that recognise native triple helical collagen and grow at a rate of several μm per hour and often, but not exclusively, radiate from locations of existing dense collagen accumulations. Some static electron tomography studies have observed fibrils extending from membrane invaginations and projections called fibripositors^33^. The authors show that following secretion, fibrils are formed at the cell surface by nucleation of collagen, just as we observe (Fig. 5A, C). The newly formed fibrils may subsequently be pulled into a membrane recess by myosin (NMII) at their membrane anchors. At this point, fibrils may grow by molecular accretion of secreted collagen, whilst tension is continually exerted by NMII. The same EM study identified that fragmented or unanchored fibrils can be internalised in a process requiring both NMII and dynamin to form fibricarriers^33^. Fibripositors have only been observed in embryonic tendon cells where linear organisation of collagen is required, with ssTEM or SBF-SEM and were not observed in our live system. In the future, our imaging platform could be used to understand whether tendon cells determine the dynamics of fibripositor formation.

We are the first to observe growing fibrils interacting with each other beneath a live cell, joining tip to tip or meeting and then growing in parallel zippering-type activity (Fig. 7). Furthermore, bundles can bifurcate or yield Y shape organization. In agreement with the appearance of fibrils in our study, this behavior has been observed in EM 3D reconstruction studies where fibrils have been seen to interact tip-to-tip, with the end of fibrils fusing together or through tip-to-shaft interactions^12,34,35^*. *In vitro studies have also shown that ends of fibrils can fuse together^12^ whilst examination of individual collagen fibrils from the skin of 12-week-old mice has identified branched networks of collagen fibrils^12^. Together, EM data of native collagen verifies that the collagen fibril behaviors reported here are physiological and are not caused by steric hindrance of the fusion protein.

Collagen bundling and higher organisation is controlled by fibril-associated collagens (FACITs) such as Col V and Small Leucine Rich Proteoglycans (SLRPs) such as decorin, expressed in Saos-2 cells^36^. These SLRPs decorate fibrils and regulate and limit the diameter of fibril bundles in tissues via their glycosaminoglycan chains which wrap around the fibril^1^. They are also insulators that prevent fibril–fibril surface interactions which can lead to fusion^37^. Knockouts of decorin or fibromodulin show increased branching of collagen networks^12^. We observed fibrils growing towards each other, sliding past and bundling, presumably creating antiparallel and parallel fibres (Figs. 7E, 9A). EM analysis of tissues has shown that they can contain both polar and unipolar fibril bundles^12^. Importantly, we have observed dynamic joining together of fibrils that evolve as the network develops, enabling the critical long-range transduction of force.

The increase in mNG-Col1α2 fluorescence at nodes or junction points to create distinct dark foci is interesting and may identify a previously unobserved structural reinforcing of crossover points of fibrils. Within this zone there is accumulation of collagen, potentially as short fibrils which interact with the existing fibril either directly or via other fibril-associated proteins. The composition of these nodes and their interactions with cells merit further study.

Cells can remodel existing collagen fibril networks to transmit forces and communicate with other cells by exerting stress on nearby fibrils driven by the actin/myosin cytoskeleton^38^. In timelapse movies, we observe existing curved fibrils straightening. Stretching of collagen along membrane extensions has previously been observed^32^. Straightening of fibrils may result from mechanical force from neighboring cells, cell migration, fibril growth or membrane expansion between fixed points. In (Fig. 9B) the fibril appears to have two connection points with the cell which hold onto the fibril as it straightens, creating right angle kinks. Such fibril to cell surface connections are likely to be established by integrins which can bind directly to collagen, such as α1β1 in the Saos-2 cell line^39^ or indirectly via collagen-integrin bridging molecules such as fibronectin^40–43^. Local exertion of force onto fibronectin can promote fibronectin fibrillogenesis, consequently facilitating local collagen assembly at these sites^31,32^. Which of these mechanisms are at play will depend on the integrin repertoire of different cell types.

In this study we show the range and possibilities of deposition dynamics and characteristics at the cellular level in a 2D cell culture environment. It is possible that not all the observed phenomena may be prevalent in vivo, where that cell is surrounded by neighboring cells and pre-existing matrix. For example, loops may be a feature of the nascent fibrils, that are not yet stabilised by covalent crosslinking by FACITs and SLRPs, and are initially highly flexible. This work provides the technological advance to test fundamental concepts and collagen deposition mechanisms in 3D model systems and tissues.

In conclusion, coupling an mNG-Col1α2 fusion protein with advanced imaging technologies has enabled us to reveal the molecular detail of collagen secretion and fibril production by living cells, providing novel and fundamental insights into the dynamic mechanisms for ECM assembly and organisation of the local collagen network. These observations shed light on how interactions of the newly secreted collagen link to both the cell and other collagen fibrils enable construction and regulation of network complexity at the cellular level. Translating this approach to different tissue types and collagen types will further our understanding of the importance of ECM composition, organisation and stiffness in aging and disease.

## Methods

### Cell lines

The human fibrosarcoma cell line HT1080 (ATCC, CCL-121™) was maintained in Dulbecco’s modified Eagle’s medium (DMEM). The primary human osteogenic sarcoma cell line Saos-2 (ECACC, 89050205) was maintained in McCoy′s 5A modified medium. Dermal BJ Fibroblasts (gifted by Procter & Gamble) were maintained in minimum Eagle’s medium (MEM). All cell lines were supplemented with 10% fetal bovine serum (FBS), 100 µgml^−1^ penicillin, 100 µgml^−1^ streptomycin and 2 mM glutaMAX and were maintained at 37 °C and 5% CO_2_. Cells were sub-cultured twice weekly.

### Transfection of cells with mNG-Col1α2

The GFP-Col1α2 published by Dallas et al. (2018) was modified to substitute mNG (237aa 26.6 kDa) as the fluorescent protein tag and include the CMV promoter to drive transcription. The construct was synthesized by VectorBuilder (VectoBuilder.com) using the pRP[Exp] vector backbone^44^. HT1080 cells were transfected using the jetPEI DNA transfection reagent (Polyplus-transfection SA) when cells reached 50–70% confluency. A 4:1 ratio of jetPEI to plasmid DNA (w/w) was diluted in NaCl following the manufacturer’s instructions and added to DMEM growth media. Imaging was conducted 24 h post-transfection. Saos-2 and BJ fibroblast cells were transfected using Lipofectamine 3000 Reagent (Invitrogen, ThermoFisherScientific) when cells reached 70–90% confluency. For Saos-2, a 3:3:2 ratio (v/w/w) of Lipofectamine 3000 reagent, P3000 reagent, and plasmid DNA were diluted in OptiMEM following the manufacturers’ instructions and added to McCoy′s 5A Medium. If collagen deposition was required, cells were supplemented with 20 μM ascorbic acid immediately after transfection. For BJ fibroblasts the same component ratio and dilution was used, however the transfection was conducted in the cell suspension phase, with cells plated at 80% confluency. For collagen deposition cells were supplemented with 20 μM ascorbic acid immediately after transfection. For all cells Imaging was conducted 48 h post-transfection.

### Imaging

Live-cell confocal images were captured using a Zeiss 880 with Airyscan, oil 63x Plan Apochromat DIC II 1.4NA lens, Zeiss Zen 2.3 SP1 FP3 (Black) V. 14.0.22.201. Laser excitation 488 nm and emission BP 495–550 for mNG-Col1α2, Laser excitation 594 nm and emission LP 570 for AntiCol1/Alexa 594, and Laser excitation 633 nm and emission LP 645 for SiR-tubulin. For collagen fibril imaging, Airyscan data was processed with jDCV (joint deconvolution—an accelerated joint Richardson-Lucy algorithm) Green channel = 8 iterations and red channel = 9 iterations. This iterative method can reach a resolution of 90 nm as measured by GATTA SIM nanorulers. For vesicle tracking, images were captured every 206 ms. Airyscan settings: 2D SR typical auto Wiener filter of 2.4. Vesicle tracking along microtubules and colchicine treatment Airyscan settings 2D SR typical auto Wiener filter of microtubules (far red) 1.4, vesicles (green) 2.4. Pixel size = 40nm Long term timelapse movies were captured using an Andor Revolution XD Spinning Disk with a 100x oil UPlanSApo1.4NA lens, Andor IQ V3. Laser excitation 488 nm, emission 525/30 nm. Pixel size 0.217 µm. Images were captured at 5 min intervals. 3D-SIM images were captured using a GE Healthcare/Deltavision OMX V4 with an Olympus PlanApoN 60x oil 1.42NA , 6 Z slices at 125 nm interval, pixel size = 80 nm. TIRF images were captured using a Deltavision OMX V4 with an Olympus TIRF 60x ApoN 1.49NA/Olympus TIRF 100x UApoN 1.49NA pixel size = 48nm. Both 3D-SIM and TIRF Laser excitation 488 nm emission 528/48 nm. 3D-SIM measured average achievable resolution FWHM using a 100 nm green bead using 488 nm excitation was 115 nm. For all live-cell imaging experiments environmental controls were set to 37 ºC and 5% CO_2_.

### Stains and treatments

Microtubules were visualised by staining with 1 μM SiR-tubulin (Tebubio, SC002) for 30 min prior to imaging. BFA treatment was conducted at 100 ng ml^−1^ for 60 min prior to fixation. Colchicine treatment was conducted at 5 μM for 2 h prior to live cell imaging; if used in conjunction with SiR-tubulin, this was added in the final 30 min of colchicine treatment.

### Image analysis

#### Vesicle analysis

Collagen vesicles tracking and mean velocity analysis was conducted using Fiji TrackMate plugin^45^.

#### Colocalisation

The Fiji plugin Coloc2 was used to perform Coste’s regression to generate a 2D intensity histogram, calculate a Person’s coefficient and perform Coste’s significance test between the red and green channels of interest^46^.

### Immunofluorescence sample preparation and staining

Cells were seeded on 18 mm No. 1.5 coverslips and transiently transfected with mNG-Col1α2 by the method listed above. Following any treatments, cells were fixed in 4% PFA for 10 min and the membrane was permeabilised with 0.1% Triton X-100 for 10 min. Blocking was conducted in 2% filtered BSA diluted in PBS for 30 min. Cells were incubated with primary antibody (1:200 in 2% BSA in PBS) overnight, and secondary antibody for 1 h (1:500 in 2% BSA in PBS). Cells were stained with 40 ng/ml DAPI for 10 min before mounting in Vectashield (H-1000–10). Col1 was stained with goat anti-type1 collagen-UNLB (Southern Biotech, 1310–01), and P4HB was stained with mouse anti-P4HB [RL90] (AB2792 Abcam). The secondary antibodies were donkey anti-goat conjugated to Alexa Fluor Plus 594 (ThermoFisher Scientific, A32758) or donkey anti-mouse conjugated to Alexa Fluor 594 (Thermofisher Scientific, A21203) respectively.

### Immunoprecipitation and mass spectrometry

HT1080 cells were lysed in radioimmunoprecipitation assay (RIPA) buffer for 5 min (1% v/v Triton X-100), 50 mM Tris HCl, pH 8, 150 mM NaCl, 0.5% w/v Na-deoxycholate, 0.1% w/v sodium dodecyl sulphate (SDS), 1X PI protease inhibitors and 1x phosSTOP (Roche). Lysates were centrifuged at 16,100 g at 4 °C for 10 min. Post-nuclear supernatants were subject to immunoprecipitation. A 30% suspension of protein A sepharose beads in RIPA buffer were incubated with the mNG antibody (32F6 Chromotek) for 1 h at 4 °C. The supernatant was removed, and beads washed 3x with RIPA buffer for 5 min. Cell lysate was mixed with the beads for 1 h at 4 °C. After incubation the beads were centrifuged at 6,000 g, the supernatant discarded, and the remaining beads washed 5 times in RIPA buffer. The beads were resuspended in elution buffer (50 mM NH_4_HCO_3_, 50 mM DTT, 1% SDS) to elute the mNGCol1α2 and its interacting proteins. Proteins were digested with filter aided sample preparation (FASP) and analysed by mass spectrometry. Sample fractions (5 μg of peptides) were analysed using an ekspertTM nanoLC 425 with low micro gradient flow module (Eksigent) attached to a quadrupole Time-Of-Flight (QTOF) mass analyser (TripleTOF 6600, SCIEX) connected to a DuoSpray source (SCIEX) and a 50-micron ESI electrode (Eksigent). The samples were loaded and then washed on a TriArt C18 Capillary guard column 1/32″, 5 μm, 5 × 0.5 mm trap column (YMC). Chromatographic separation was performed over 57 min on a TriArt C18 Capillary column 1/32″, 12 nm, S-3 μm, 150 × 0.3 mm (YMC) at a flow rate of 5 μl min^−1^ with a linear gradient of 3–32% acetonitrile, 0.1% formic acid over 43 min; then to 80% acetonitrile, 0.1% formic acid over 2 min, held for 3 min before returning to 3% acetonitrile, 0.1% formic acid and re-equilibrated. Analyst software (version 1.7.1, Applied Biosystems) was used to acquire the MS and MS/MS data.

## Electronic supplementary material

Below is the link to the electronic supplementary material.

Supplementary Material 1

Supplementary Material 2

Supplementary Material 3

Supplementary Material 4

Supplementary Material 5

Supplementary Material 6

Supplementary Material 7

Supplementary Material 8

Supplementary Material 9

Supplementary Material 10

Supplementary Material 11

Supplementary Material 12

Supplementary Material 13

Supplementary Material 14

Supplementary Material 15

Supplementary Material 16

Supplementary Material 17

Supplementary Material 18

Supplementary Material 19

Supplementary Material 20

Supplementary Material 21

Supplementary Material 22

Supplementary Material 23

Supplementary Material 24

Supplementary Material 25

## Data Availability

Data is provided within the manuscript or supplementary information files.
