# Supplementary material for: New imaging tools reveal live cellular collagen secretion, fibril dynamics and network organisation
Source: Sci Rep. 2025 Apr 21;15:13764. doi: 10.1038/s41598-025-96280-4 (PMC12012225; doi:10.1038/s41598-025-96280-4)
Supplement: Supplementary file 1 — Supplementary Material 1 [file 41598_2025_96280_MOESM1_ESM.docx]

**Supplementary Information**

**Kent *et al.* 2024**

**New imaging tools reveal live cellular collagen secretion, fibril dynamics and network organisation.**

**Supplementary Figure 1:** Assessment of the binding partners, processing and trafficking of the mNG-Col1α2 fusion protein.

**(A)** Table of mNG-Col1α 2 interacting proteins identified by mNG IP followed by DDS MS. **(B)** Schematic diagram showing the location of hydroxylation within the mNG-Col1α2 polypeptide as analysed by mNG IP followed by DDS MS. **(C)** Immunofluorescence confocal imaging of mNG-Col1α2 (green) expressing HT1080 cells, co-labeled for the ER marker and collagen processing chaperone P4HB (PDIA1) (red) and DAPI. **(D)** Same staining conditions as C but following BFA treatment. Following BFA treatment the mNG-Col1α2 signal is retained in the ER. To the right are graphical representations of the Pearsons colocalization correlation for each image set. Scale = 20 μm.

**Supplementary Figure 2:** BFA treatment of Saos-2 cells expressing the mNG-Col1α2 fusion protein****

**(A)** Immunofluorescence confocal imaging of mNG-Col1α2 (green) expressing Saos-2 cells, co-labeled for the ER marker and collagen processing chaperone P4HB (PDIA1) (red) and DAPI. **(B)** Same staining conditions as A but following BFA treatment. Following BFA treatment the mNG-Col1α2 signal is retained in the ER. Scale = 10 μm.

**Supplementary Figure 3:** BJ Fibroblast cells expressing the mNG-Col1α2 fusion protein.

Two example fibroblast cells show motile collagen carriers and deposit mNG-Col1α2 containing collagen fibrils, comparable to those seen in Saos-2 cells. Scale = 20 μm.

**Supplementary Movie 1:** Movement of mNG-Col1α2 containing carriers in HT1080 cells. Analysis of mNG-Col1α2 labelled collagen carriers which move at high speeds, predominately radiating towards the cell periphery. Images were acquired every 206ms with a total of 160 frames.

**Supplementary Movie 2:** Trackmate analysis of mNG-Col1α2 carriers**.** Video of mNG-Col1α2 containing carriers in HT1080 cells (as in supplementary movie 1) with Trackmate tracks overlaid. Trackmate analysis enabled visualisation of carrier paths and quantification of individual velocities. Images were acquired every 206ms with a total of 160 frames.

**Supplementary Movie 3:** mNG-Col1α2 containing carriers associate with and travel along microtubules. Images were acquired every 8.66 seconds for a total of 10 frames.

**Supplementary Movie 4:** mNG-Col1α2 movement along microtubules colchicine control. mNG-Col1α2 containing carriers associate with and travel along microtubules towards the cell periphery. Microtubules (white), mNG-Col1α2 (green). Object detection yellow ring with red tracks. Images were acquired every 206.44 ms per channel (412.88ms) for a total of 60 frames.

**Supplementary Movie 5:** Microtubule disruption by colchicine stops mNG-Col1α2 containing carrier movement**.** Following colchicine treatment intact microtubules are no longer visible and mNG-Col1α2 containing carriers remain stationary. Microtubules (white), mNG-Col1α2 (green). Object detection yellow ring with red tracks. Images were acquired every 206.44 ms per channel (412.88ms) for a total of 60 frames.

**Supplementary Movie 6:** **mNG-Col1α2 secretion from HT1080 cells.** Video of a single mNG-Col1α2 fusion protein containing carrier. Movement occurs towards the PM where it remains stationary before fusing and releasing contents. Images were acquired every 10 ms or 100 fps.

**Supplementary Movie 7:** Video of the movement of mNG-Col1α2 containing carriers in Saos-2 cells with Trackmate tracks overlaid. Images were acquired every 206ms with a total of 160 frames.

**Supplementary Movie 8: mNG-Col1α2 particles in Saos-2 cells move along microtubules.** Video of mNG-Col1α2 containing carriers in Saos-2 cells co-stained for microtubules with SiR-tubulin. Trackmate tracks of each carrier are overlaid. Images were acquired every 410ms with a total of 45 frames.

**Supplementary Movie 9: mNG-Col1α2 particles in Saos-2 cells move along microtubules.** A second example Video of mNG-Col1α2 containing carriers in Saos-2 cells co-stained for microtubules with SiR-tubulin. Trackmate tracks of each carrier are overlaid. Images were acquired every 1.63 s with a total of 30 frames.

**Supplementary Movie 10:** Saos-2 cells. mNG-Col1α2 movement along microtubules, colchicine control. mNG-Col1α2 containing carriers associate with and travel along microtubules towards cell periphery. Microtubules (white), mNG-Col1α2 (green). Object detection yellow ring with red tracks. Images were acquired every 206.44 ms per channel (412.88ms) for a total of 60 frames.

**Supplementary Movie 11:** Saos-2 cells. MT disruption by colchicine stops mNG-Col1α2 containing carrier movement**.** Following colchicine treatment intact microtubules are no longer visible and mNG-Col1α2 containing carriers remain stationary. Microtubules (white), mNG-Col1α2 (green). Object detection yellow ring with red tracks. Images were acquired every 206.44 ms per channel (412.88ms) for a total of 60 frames.

**Supplementary Movie 12:** mNG-Col1α2 fibril bleaching. When a mNG-Col1α2 fibril is exposed to continuous laser exposure the signal does not bleach uniformly, reflecting the number of mNG-Col1α2 proteins and their distribution within the fibril. 10 frames per minute.

**Supplementary Movie 13:** Collagen fibril linear growth. Video of the linear and directional growth of a mNG-Col1α2 containing collagen fibril. Frame rate is 1 frame acquired every 5 minutes or 12 frames per hour.

**Supplementary Movie 14: Collagen fibril growth along an existing fibril.** This video shows an initial faint collagen fibril grow and enter the frame. There are two clear growths of a new fibril along the pre-existing fibril or path. As each fibril extends the mNG-Col1α2 intensity increases in a directional manner. Frame rate is 1 frame acquired every 5 minutes or 12 frames per hour.

**Supplementary Movie 15:** Collagen fibril bifurcation, zippering and bundling**.** Video shows a collagen fibril grow from top to bottom of the frame. In later time points this singular fibril splits into two thinner fibrils which continue to grow in the original direction. In the centre of the frame two fibrils grow in opposite directions before meeting and bundling together. They zipper up from the bottom of the frame upwards. Finally, the remaining fibril to the right snaps across in just 4 frames to join with the fibril on the left. Frame rate is 1 frame acquired every 5 minutes or 12 frames per hour.

**Supplementary Movie 16:** Collagen fibril cross over and fluorescence intensity maxima. In this video several collagen fibrils grow across the frame in different directions. Dark punctate maxima of mNG-Col1α2 signal can be seen developing on a fibril at the approximate location at which another fibril will cross over it. Once these crossover points or nodes are established the mNG-Col1α2 maxima continue to grow in size and intensity. Frame rate is 1 frame acquired every 5 minutes or 12 frames per hour.

**Supplementary Movie 17:** Collagen fibril meeting. In the video two mNG-Col1α2 containing collagen fibrils grow in opposite directions towards each other. As they meet they continue to grow in their original direction but now along the opposing fibril. Frame rate is 1 frame every aquired 5 minutes or 12 frames per hour.

**Supplementary Movie 18:** Collagen fibril straightening. In this video a large curve of collagen straightens into a square U shape. The fibril appears to be attached at two points between which the curve is straightened into a series of straight lines with effective right angled corners. The movie is an example of a live cell potentially imparting mechanical force on its surrounding collagen network, either directly or during cell migration. Alternatively, this could also result from fibril growth between these points. Frame rate is 1 frame acquired every 5 minutes or 12 frames per hour.

**Supplementary Movie 19:** Collagen fibril growth into loops**.** In this movie an mNG-Col1α2 containing fibril grows and curls back on itself generating a loop. This loop then increases in thinness and intensity as new collagen fibrils grow along this looped fibril/path. Frame rate is 1 aquired frame every 5 minutes or 12 frames per hour.

**Supplementary Movie 20:** Collagen fibril looping. In this video an existing collagen fibril is moved by the cell or flow of culture media to loop up around itself. Frame rate is 1 frame acquired every 5 minutes or 12 frames per hour.

**Supplementary Movie 21:** Collagen fibril interwinding**.** This video shows two separate collagen fibrils growing along paths that wrap around each other and intertwine the two strands. Frame rate is 1 frame every 5 minutes or 12 frames per hour.
