# Supplementary figures and images for: New imaging tools reveal live cellular collagen secretion, fibril dynamics and network organisation
Source: Sci Rep. 2025 Apr 21;15:13764. doi: 10.1038/s41598-025-96280-4 (PMC12012225; doi:10.1038/s41598-025-96280-4)

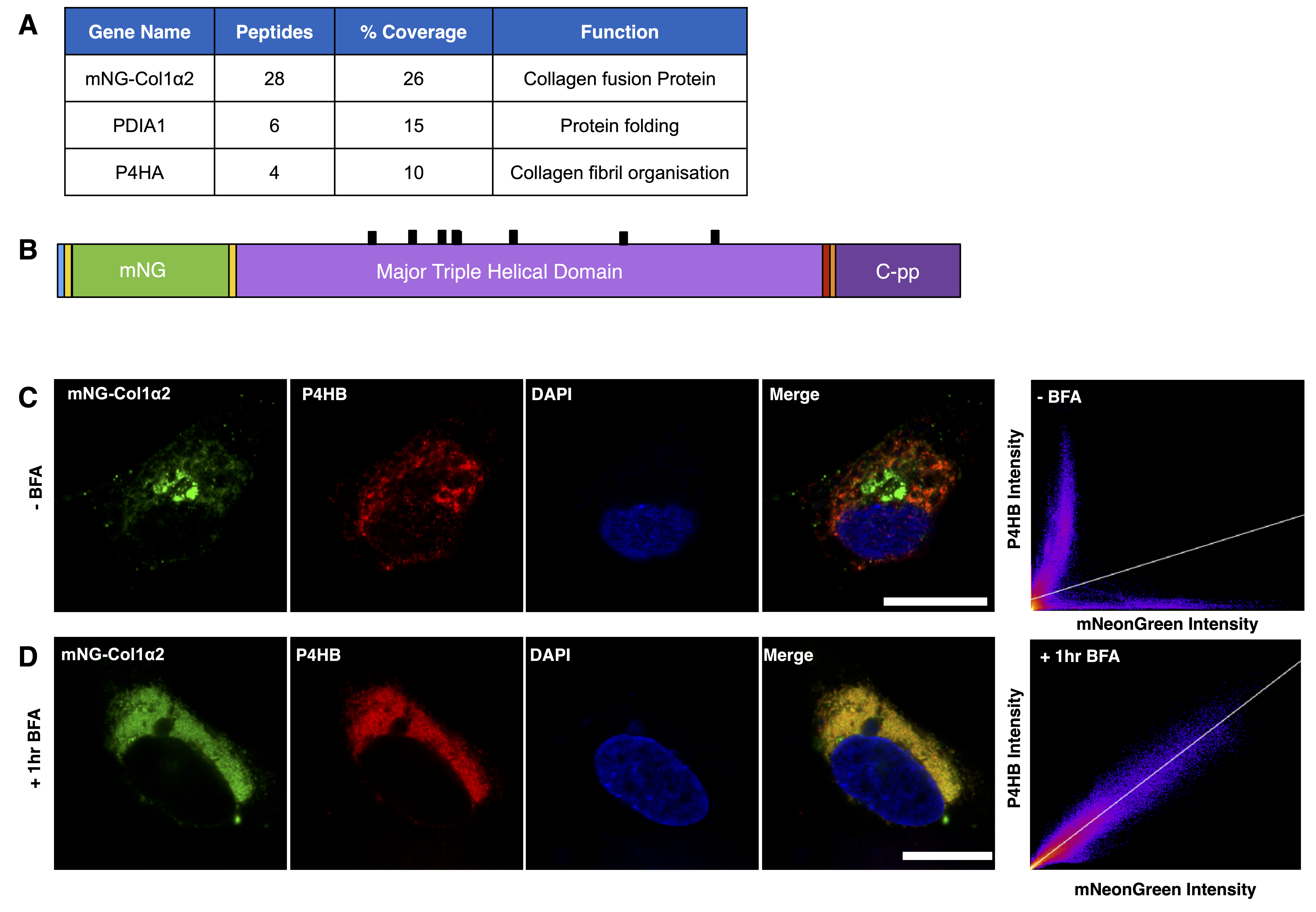

Supplement: Supplementary file 2 — Supplementary Material 2 [file 41598_2025_96280_MOESM2_ESM.tiff]

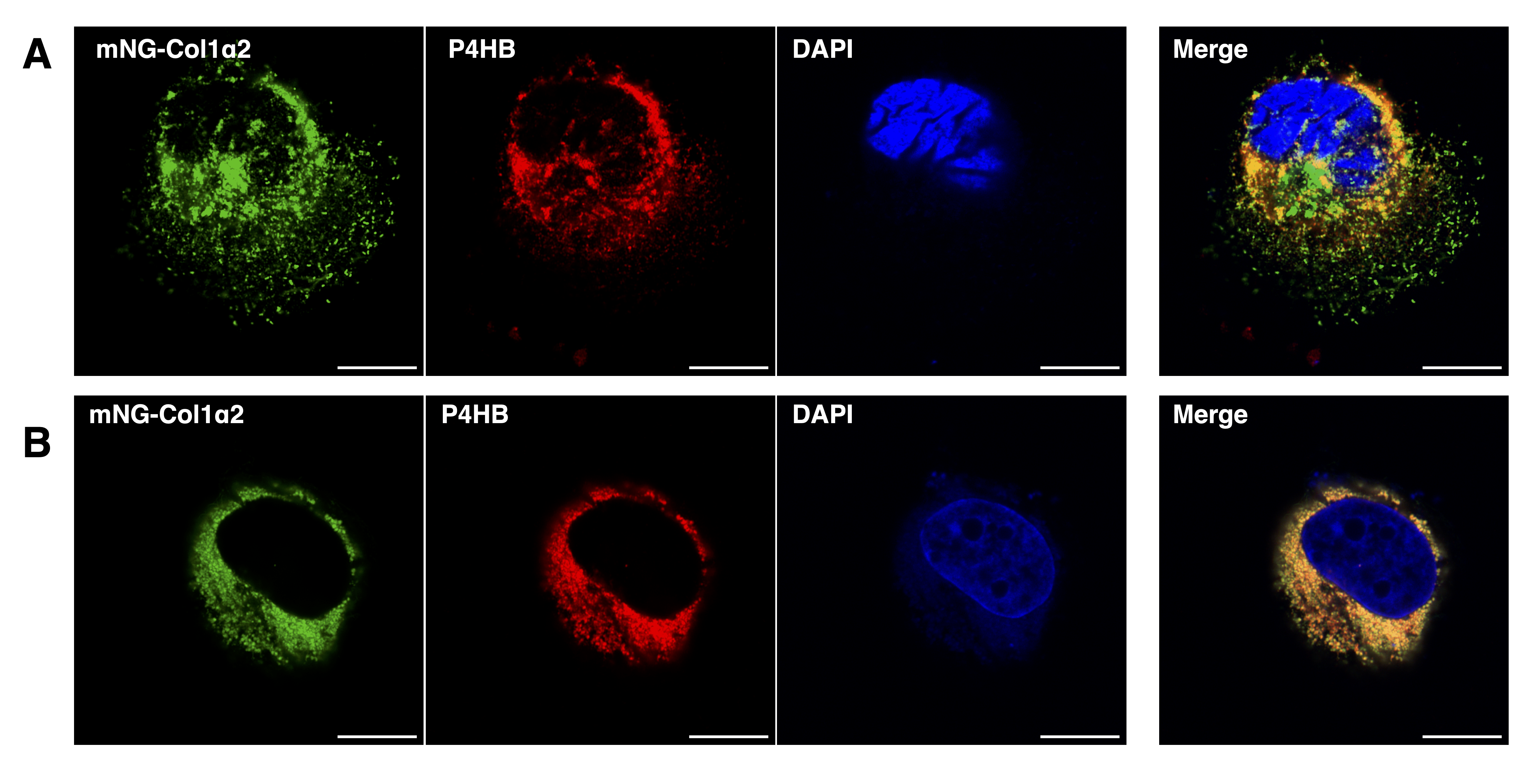

Supplement: Supplementary file 3 — Supplementary Material 3 [file 41598_2025_96280_MOESM3_ESM.tiff]

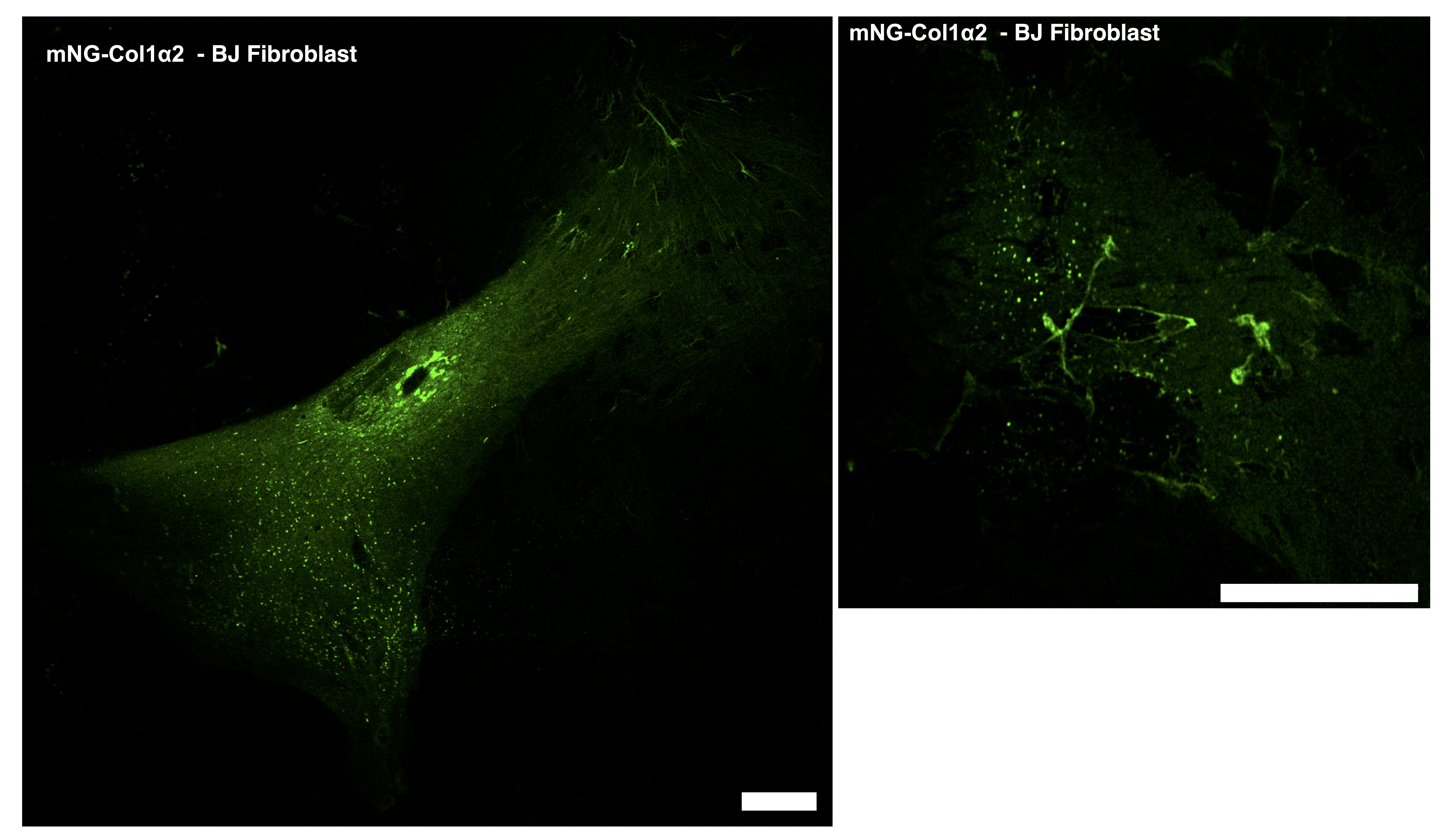

Supplement: Supplementary file 4 — Supplementary Material 4 [file 41598_2025_96280_MOESM4_ESM.tiff]
